# Quantitative Analyses Reveal How Hypoxia Reconfigures the Proteome of Primary Cytotoxic T Lymphocytes

**DOI:** 10.3389/fimmu.2021.712402

**Published:** 2021-09-17

**Authors:** Sarah H. Ross, Christina M. Rollings, Doreen A. Cantrell

**Affiliations:** ^1^Immunology Programme, The Babraham Institute, Cambridge, United Kingdom; ^2^Division of Cell Signalling and Immunology, School of Life Sciences, University of Dundee, Dundee, United Kingdom

**Keywords:** hypoxia, oxygen sensing, CTLs, cytotoxic lymphocytes, quantitative proteomics, CD8 T cells

## Abstract

Metabolic and nutrient-sensing pathways play an important role in controlling the efficacy of effector T cells. Oxygen is a critical regulator of cellular metabolism. However, during immune responses T cells must function in oxygen-deficient, or hypoxic, environments. Here, we used high resolution mass spectrometry to investigate how the proteome of primary murine CD8^+^ cytotoxic T lymphocytes (CTLs) is reconfigured in response to hypoxia *in vitro*. We identified and quantified over 7,600 proteins and discovered that hypoxia increased the abundance of a selected number of proteins in CTLs. This included glucose transporters, metabolic enzymes, transcription factors, cytolytic effector molecules, checkpoint receptors and adhesion molecules. While some of these proteins may augment the effector functions of CTLs, others may limit their cytotoxicity. Moreover, we determined that hypoxia could inhibit IL-2-induced proliferation cues and antigen-induced pro-inflammatory cytokine production in CTLs. These data provide a comprehensive resource for understanding the magnitude of the CTL response to hypoxia and emphasise the importance of oxygen-sensing pathways for controlling CD8^+^ T cells. Additionally, this study provides new understanding about how hypoxia may promote the effector function of CTLs, while contributing to their dysfunction in some contexts.

## Introduction

CD8^+^ cytotoxic T lymphocytes (CTLs) are essential adaptive immune cells that destroy pathogen infected cells and tumour cells. CD8^+^ T cell differentiation is directed by antigen receptors and signalling pathways co-ordinated by co-stimulatory receptors, inhibitory receptors and cytokines (1). Environmentally controlled metabolic signalling pathways are also critical regulators of CTL differentiation and function (2–4), and as CD8^+^ T cells respond to immune activation they increase expression of nutrient transporters, nutrient sensors, and oxygen-sensing molecules (5).

Oxygen (O_2_), is essential for oxidative phosphorylation, and the energy production required to sustain metazoan cells (6). However, the oxygen environment that T cells experience *in vivo* can be quite varied, and T cells have even been found in hypoxic environments, i.e. a site where there is a deficiency in oxygen caused by an imbalance between oxygen supply and demand. For example, lymph node germinal centres (7) and the gut mucosal barrier of the intestine (8) are relatively hypoxic. Additionally, viral infections can trigger localised hypoxia in lung tissues because of damage to alveolar epithelial cells and pulmonary capillaries (9, 10). An insufficient blood supply due to poor or damaged vasculature can also contribute to tissue hypoxia: it is well established that the suboptimal architecture of blood vessels in tumours contributes to a hypoxic microenvironment (11–13). Indeed, T cells have been observed within hypoxic areas of some tumours (14–17).

Studies have examined how changes in oxygen availability impact on CD8^+^ T cells. Exposure of CD8^+^ T cells to hypoxia, either during the initial priming of naïve T cells or once they had differentiated into effector CTLs, has been shown to enhance CD8^+^ T cell cytolytic capacity (18–23), inhibit T cell proliferation (18, 22–25), and increase the expression of the IL-2 receptor alpha chain (IL-2Rα, also known as CD25) (18, 22), costimulatory receptors (22, 26, 27) and inhibitory checkpoint molecules (22, 23, 26, 27) on CD8^+^ T cells. Hypoxic CD8^+^ T cells have been reported to increase expression of effector molecules (26–28) and the production of cytokines such as IL-10 (22, 23) and Interferon gamma (IFNγ) (26, 29, 30). However, in this latter context there are also some conflicting data that hypoxia decreases IFNγ production (16, 18, 31) by T cells.

One important oxygen-sensing pathway in CTL is mediated by HIF1 (hypoxia-inducible factor 1) transcriptional complexes (26–28, 32–34). The expression of the HIF1alpha (HIF1α) subunit of the HIF1 complex is regulated by the oxygen-dependent PHD family prolyl hydroxylases (PHD1,2 3) (6, 35, 36). In high oxygen, PHD proteins hydroxylate prolines in HIF1α targeting it for ubiquitylation mediated by the VHL-containing ubiquitin ligase complex and subsequent proteasomal degradation. HIF1α levels are thus lower in CTLs when environmental oxygen is high but rapidly increase in response to reduced oxygen availability. Loss of VHL or PHDs is linked to enhanced effector CD8^+^ T cell function (27, 32, 33). There are, however, HIF- independent ways whereby changes in oxygen availability could impact T cells. CTLs still depend on oxidative phosphorylation *in vivo* (37) and PHDs can have substrates other than HIF1α (38, 39). There are also histone demethylases which utilize oxygen in their catalytic mechanism to couple environmental oxygen changes to epigenetic histone modifications (40–42). Additionally, oxygen control of mRNA translation (43) and pathways of protein degradation are part of cellular responses to changing oxygen availability. For example, CTLs express the cysteine dioxygenase ADO which controls the oxygen-dependent degradation of proteins *via* the N-degron pathway (44). Therefore, hypoxia may reconfigure the phenotype of T cells at multiple levels.

As hypoxia, and activation of hypoxia-induced gene expression, has been linked to both promoting and inhibiting the effector function of CTLs, the objective of this study was to examine the impact of hypoxia on pure cultures of CTLs differentiated *in vitro*. We used mass spectrometry-based proteomics to perform a high-resolution unbiased analysis of the proteome of CTLs in normoxia and hypoxia. These data provide a comprehensive understanding of how hypoxia alters the proteomic landscape of CTLs and provides new insights towards reconciling how hypoxia may promote CTL effector function, yet lead to their dysfunction in different contexts.

## Materials and Methods

### Mice

P14 mice have been described previously (45). Female P14 T cell receptor transgenic mice were used for proteomic studies, and both male and female mice were used for other experiments. All mice were bred and maintained in the University of Dundee in compliance with UK Home Office Animals (Scientific Procedures) Act 1986 guidelines.

### *In Vitro* Cultures of Cytotoxic T Lymphocytes (CTLs)

CTLs were generated as described previously (34). Briefly, P14 CD8^+^ naïve T cells taken from the spleens of mice were activated by triggering of the T cell receptor (TCR) with 100 ng/ml gp-33 peptide of the Lymphocytic Choriomeningitis Virus (LCMV) for 48 hours in the presence of 20 ng/ml IL-2 (Proleukin, Novartis) and 2 ng/ml IL-12 (Peprotech) in a humidified incubator with a gas atmosphere of 18% oxygen and 5% CO_2_.

After 48 hours, the activated CD8^+^ cells were removed from TCR stimulation and were then cultured in RPMI 1640 (ThermoFisher Scientific), with 10% foetal bovine serum (FBS) (Life Technologies), supplemented with 50 units/ml penicillin-G (GIBCO), 50 μg/ml streptomycin (GIBCO), 50 μM β-mercaptoethanol (Sigma-Aldrich) and 20 ng/ml IL-2 (Proleukin, Novartis) in a gas atmosphere of 18% oxygen and 5% CO_2_. Cells were counted every day, and split to a density of 5×10^5^ cells per ml in RPMI 1640 with 10% FBS, 50 units/ml penicillin-G, 50 μg/ml streptomycin and 50 μM β-mercaptoethanol with fresh IL-2 being added at a final concentration of 20 ng/ml. Samples were considered biological replicates if CTLs were generated from separate spleens.

For most of this study, we analysed the impact of hypoxia on CTLs after 24 hours: specifically, for the proteomics analyses, flow cytometry forward and side scatter analyses, lactate production, protein synthesis assays, cell cycle analyses and determination of cytokine production after antigen-receptor triggering. For all of these experiments, CD8^+^ T cells were differentiated in IL-2 for 4 days in a gas atmosphere of 18% oxygen and 5% CO_2_ before being subjected to hypoxia as detailed in “24-hour hypoxia treatments”. For proliferation assays, we counted cells over 48 hours in hypoxia. To ensure that the cells were still undergoing exponential growth during the evaluation, CD8^+^ T cells were differentiated in IL-2 for 3 days in a gas atmosphere of 18% oxygen and 5% CO_2_ before being subjected to hypoxia as detailed in “48-hour proliferation assays”.

### Flow Cytometry for FSC/SSC, Viability and Cell Counting

For routine culture, and for setting up specific analyses, suspensions of CD8 T cells in RPMI were taken directly from the cell cultures and were diluted 1:10 with 1% FBS (v/v) in PBS + DAPI. FSC and SSC data and DAPI signal were acquired on a FACSVerse flow cytometer with FACSuite software (BD Biosciences). Viable cells were determined according to DAPI staining. The volumetric measurements acquired by the FACSVerse during sample acquisition were used to determine an accurate cell count. Data analysis was performed with FlowJo software (Treestar).

### 24-Hour Hypoxia Treatments

Following activation and 4 days of differentiation in IL-2, CTLs were counted and resuspended at a concentration of 3×10^5^ cells per ml in RPMI 1640 with 10% FBS, 50 units/ml penicillin-G, 50 μg/ml streptomycin, 50 μM β-mercaptoethanol and 20 ng/ml IL-2. Cells were seeded at 3×10^5^ cells/ml to mitigate for nutrient deprivation that may occur in hypoxia through a combination of proliferation, and the upregulation of nutrient transporters such as GLUT1 and GLUT3. A volume of 8 mls of the cell suspension was plated per well of a 6 well plate. Cells were rested for 2 hours before being maintained in a gas atmosphere of 18% oxygen and 5% CO_2_ or being transferred to an atmosphere of 1% O_2_ and 5% CO_2_ in a Galaxy 48 R incubator (Eppendorf) for 24 hours. After 24 hours, cells were counted and processed based on the requirements of individual experiments.

### 48-Hour Proliferation Assays

For proliferation assays, CTLs that had been generated by activating naïve CD8^+^ T cells for 2 days followed by differentiation in IL-2 for 3 days were counted and split to 3×10^5^ cells per ml in RPMI 1640 (plus 10% FBS, 50 units/ml penicillin-G, 50 μg/ml streptomycin, 50 μM β-mercaptoethanol) with 20 ng/ml IL-2. A volume of 8 mls of the cell suspension was plated per well of a 6 well plate. Cells were either maintained at 18% oxygen + 5% CO_2_ or transferred to an atmosphere of 1% O_2_ + 5% CO_2_ in a Galaxy 48 R incubator (Eppendorf). After 24 hours, cells were counted to evaluate cell proliferation. Additionally, these counts were used to split cells, in duplicate plates, to 3×10^5^ cells per ml with fresh IL-2 being added at a final concentration of 20 ng/ml. For these experiments, the RPMI was pre-equilibrated to 18% or 1% oxygen by keeping aliquots of the media in plates in the appropriate incubators overnight. Cells were counted 24 hours later to generate the data for the 48 hour time point.

### Protein Digestion for Proteomic Samples

Pellets of CTLs prepared as described in “24-hour hypoxia treatments” were lysed in 400 μl lysis buffer (4% (v/v) sodium dodecyl sulphate (SDS), 50 mM tetraethylammonium bromide (TEAB) pH 8.5 and 10 mM tris(2-carboxyethyl)phosphine hydrochloride (TCEP)), and incubated at 22°C under agitation (500 rpm on Thermomixer) for 5 minutes. Following the incubation, the lysates were boiled for 5 minutes under agitation (500 rpm on Thermomixer). The lysates were cooled and sonicated for 15 cycles of 30-seconds-on and 30-seconds-off using a UP200St with VialTweeter (Hielscher Ultrasound Technology) without cooling. The concentration of proteins in the cell lysates was determined using an EZQ protein quantitation kit (Invitrogen) as per manufacturer instructions. Following protein quantitation, the samples were incubated with 20 mM iodoacetamide for 1 hour at 22°C in the dark to alkylate reduced cysteines.

Paramagnetic beads were used for protein clean-up and digest according to the Single-Pot Solid-Phase-enhanced Sample Preparation (SP3) procedure (46). Briefly, 200 μg of a 1:1 mix of Hydrophobic and Hydrophilic Sera-Mag SpeedBead Carboxylate-Modified Magnetic Particles were added to each protein sample. The samples were acidified by the addition of 550 μl 10:1 acetonitrile:formic acid. The samples were then incubated at 22°C under agitation (500 rpm on Thermomixer) for 8 minutes to allow the proteins to bind to the beads. The bead-protein complexes were immobilized using a magnetic rack and the supernatant was removed before washing the complexes once with 70% (v/v) ethanol and then once with acetonitrile. After air drying, the beads were resuspended in digest buffer (0.1% (v/v) SDS; 50 mM TEAB, pH 8.5; 1 mM CaCl_2_). LysC (Wako) was added at a ratio of 50:1 protein:LysC and incubated for 16 hours at 37°C. Then Trypsin (Promega) was added at a ratio of 50:1 protein:Trypsin, and left for 24 hours at 37°C. After the incubation with the proteases, acetonitrile was added to the digest mix, to give a final concentration of >95% (v/v) acetonitrile. The samples were mixed and incubated upright for 8 minutes without agitation, followed by incubation for 2 minutes on a magnetic rack to immobilise the beads. The acetonitrile/digest buffer mix was removed, and the bead-peptide complexes were washed using acetonitrile. To elute the peptides, 2% (v/v) DMSO was added to the beads before sonication (5 cycles of 30 seconds on and 30 seconds off, without cooling). The supernatant containing the peptides was removed into a fresh tube. Formic acid was added to the peptides to give a final concentration of 5% (v/v) formic acid.

### Peptide Fractionation

Peptide samples for proteomic analysis were fractionated off-line by high pH reverse-phase liquid chromatography using an UltiMate 3000 BioRS system equipped with a 2.1 mm × 150 mm Xbridge Peptide BEH C18 column with 3.5 μm particles (Waters). For the separation, the buffers used were 10 mM ammonium formate at pH 9 in 2% (v/v) acetonitrile (RPLC buffer A) and 10 mM ammonium formate at pH 9 in 80% (v/v) acetonitrile (RPLC buffer B). The samples were separated using a 25 minute multistep gradient of RPLC buffer A and RPLC buffer B at a flow rate of 0.3 ml per minute. Peptides were separated into 16 fractions using a concatenation collection method. The fractions were dried using a SpeedVac (Genevac).

### Liquid Chromatography-Mass Spectrometry

Mass spectrometry was performed by the Proteomics Facility, University of Dundee, UK. Analysis of peptides was performed on a Velos Pro Orbitrap mass spectrometer (Thermo Scientific) coupled with a Dionex Ultimate 3000 RS (Thermo Scientific). The liquid chromatography (LC) buffers were LC buffer A (0.1% (v/v) formic acid in Milli-Q water) and LC buffer B (80% (v/v) acetonitrile, 0.08% (v/v) formic acid in Milli-Q water).

Dried peptide samples were reconstituted in 50 µl of 1% (v/v) formic acid and 7 μl of each sample were loaded at 10 μl/min onto a trap column (100 μm × 2 cm, PepMap nanoViper C18 column, 5 μm, 100 Å, Thermo Scientific) equilibrated in LC buffer A for 19 minutes. The trap column was washed for 3 minutes at the same flow rate before the trap column was switched in-line with a Thermo Scientific resolving C18 column (75 μm × 50 cm, PepMap RSLC C18 column, 2 μm, 100 Å) kept at a constant temperature of 50°C and equilibrated in 2% LC buffer B for 19 minutes. Peptides were eluted from the column at a constant flow rate of 300 nl/minute with a linear gradient from 2% LC buffer B to 5% LC buffer B within 3 minutes, then from 5% LC buffer B to 35% LC buffer B in 124 minutes and finally from 35% LC buffer B to 98% LC buffer B in 2 minutes. The column was then washed for 20 minutes at 98% LC buffer B and re-equilibrated in 2% LC buffer B for 19 minutes. The LTQ Orbitrap Velos Pro was operated in data dependent positive ionization mode using an Easy-Spray nanoelectrospray ion source (Thermo Scientific). The source voltage was set to 1.9 Kv and the capillary temperature was 250°C. A scan cycle comprised MS1 scan (m/z range from 335-1800) in the Velos Pro Orbitrap followed by 15 sequential dependant MS2 scans (the threshold value was set at 5,000 and the minimum injection time was set at 200 ms) in the LTQ (Linear Trap Quadrupole) with collision induced dissociation using the following parameters: isolation width 2, normalized collision energy 35, default charge state 2, activation Q 0.25 and activation time, 10 ms. The resolution of the Orbitrap Velos was set to 60,000 after accumulation of 1,000,000 ions. Precursor ion charge state screening was enabled, with all unassigned charge states and singly charged species rejected. The lock mass option was enabled for survey scans to improve mass accuracy. To ensure mass accuracy, the mass spectrometer was calibrated on the first day that the runs were performed.

### Proteomic Data Processing and Analysis

The mass spectrometry data files were processed using MaxQuant version 1.6.0.1 and spectra were mapped to the reviewed UniProtKB mouse protein database and the contaminant database supplied by MaxQuant. The following search parameters were used: trypsin and LysC were selected as the proteases; up to two missed cleavages were permitted; the minimum peptide length was set to 6 amino acids; protein N-terminal acetylation, methionine oxidation, glutamine to pyroglutamate, glutamine and asparagine deamidation were selected as variable modifications; carbamidomethylation of cysteine residues was set as a fixed modification; MS tolerance of 20 ppm and MS/MS tolerance of 0.5 Da; label free quantification was enabled. False discovery rates (FDRs) were set to 0.01 and based on hits against the reversed sequence database. This cut-off was applied to individual spectra and whole proteins in the MaxQuant output. The match between runs function was enabled. Proteins were quantified on the basis of unique (found only in a specific protein group) and razor peptides (peptides assigned to a specific protein group without being unique to that group) with the re-quantification feature enabled.

Following processing of the files by MaxQuant, Perseus software version 1.5.2.6, was used to annotate proteins with gene ontology (GO) terms for biological process (BP), molecular function (MF), cellular compartment (CC), and KEGG pathways; filter the MaxQuant output to remove known contaminants, reverse sequences and proteins only identified based on a modification site; and calculate estimated copy numbers of proteins per cell.

The proteome ruler plug-in was implemented in Perseus to assign copy numbers of histones in a diploid mouse cell to the summed peptide intensities of all histones in each sample. From this, Perseus used the peptide intensities associated with the other proteins identified in the dataset to estimate their copy numbers per cell (47). The accuracy of the quantification of each protein was annotated according to the number of peptides and percentage of unique peptides identified for the protein as follows: high: ≥8 peptides detected, minimum of 75% unique peptides; medium: ≥3 peptides detected, a minimum of 50% unique peptides; low: all other peptides.

To evaluate changes to the CTL proteome induced by hypoxia, the estimated copy numbers were used to determine the ratio of expression of individual proteins within biological replicates. The significance of these changes was determined by performing a two-tailed paired Student’s t-test, without further adjustment, on Log2-normalized copy numbers for each protein in normoxia and hypoxia using Microsoft Excel. Proteins were defined as significantly regulated if they had a ratio of ≥1.5 or ≤ 0.67, if they were identified in two out of three replicates, if they were reproducibly regulated in all replicates in which they were identified, and if they had a P value of ≤0.05.

The proteins identified as being up- or down-regulated were subjected to enrichment analysis for KEGG pathways, using a custom background comprising of all proteins identified in the dataset. The enriched KEGG pathways were grouped using the functional annotation clustering tool of DAVID (version 6.8), using a medium clustering stringency and an EASE (Expression analysis systematic explorer) score, a modified Fisher Exact P value, of ≤1.

Copy numbers were additionally used to calculate the mass of specific proteins, or groups of proteins, per cell. For each protein, the following formula was used to calculate its total mass per cell: Mass of proteins (in grams per cell) = [(protein copy number)/Avogadro’s constant] × molecular weight of protein (Daltons). When specific groups of proteins were analysed, for example glycolytic enzymes, the proteins were manually filtered in Excel, based on KEGG pathway annotation by Perseus.

### Lactate Output Measurements

Lactate measurements were performed as described previously (28, 34). Briefly, CTL that had been seeded and maintained in normoxia or subjected to hypoxia for 24 hours as described in “24-hour hypoxia treatments” were counted and resuspended to a concentration of 1x10^6^ cells/ml in fresh RPMI with 10% (v/v) dialysed serum equilibrated in the appropriate oxygen atmosphere. The cells were incubated for a further 4 hours in the appropriate oxygen environment. Following incubation, the media supernatants were collected. The concentration of lactate released into the media was determined using an enzymatic assay in which lactate dehydrogenase (LDH) was used to oxidise lactate and the reduction of NAD^+^ to NADH was measured by absorption at 340 nM. To determine lactate concentration, an equal volume of sample and mastermix (320 mM glycine, 320 mM hydrazine, 2U lactate dehydrogenase and 2.4 mM NAD^+^) were mixed together and samples incubated for 10 minutes at room temperature. Absorbance of the reactions was then read at 340 nM using a cytofluor II Fluorescence Multi-Well Plate Reader (Perceptive BioSystems). Lactate standards were used to generate a standard curve and the concentration of lactate in the test samples inferred following data analysis in Prism (GraphPad).

### Protein Synthesis Analysis

CTLs that had been seeded as described in “24-hour hypoxia treatments” were counted 24 hours later and resuspended to a final concentration of 1x10^6^ cells/ml in fresh RPMI 1640 with 10% FBS, 50 units/ml penicillin-G, 50 μg/ml streptomycin, 50 μM β-mercaptoethanol and 20 ng/ml IL-2 equilibrated to the appropriate oxygen atmosphere. The CTLs were incubated with 20 µM O-propargyl-puromycin (OPP, Jena Bioscience) for 10 minutes at 37°C in normoxia (18% O_2_) or hypoxia (1% O_2_). Control cells cultured at 18% O_2_ were pre-treated with cycloheximide for 30 minutes before addition of OPP. OPP incorporation into polypeptide chains was terminated by fixing cells with 1% paraformaldehyde for 15 minutes at 22°C. After fixation, cells were washed in 0.5% (v/v) FBS in PBS before being permeabilised with 0.5% (v/v) Triton X-100 in PBS for 15 minutes at 22°C. The incorporated OPP was then labelled with Alexa 647-azide using a standard Click-IT chemistry reaction (ThermoFisher Scientific) for 30 minutes at 22°C. Cells were washed twice with 0.5% FBS (v/v) in PBS, before being resuspended in 0.5% FBS (v/v) in PBS for analysis. Data were acquired on a LSRFortessa flow cytometer with DIVA software (BD Biosciences). Data analysis was performed with FlowJo software (Treestar).

### Cell Cycle Analysis

CTLs seeded as described in “24-hour hypoxia treatments” were counted and resuspended to a final concentration of 1x10^6^ cells/ml in fresh RPMI 1640 with 10% FBS, 50 units/ml penicillin-G, 50 μg/ml streptomycin, 50 μM β-mercaptoethanol and 20 ng/ml IL-2 equilibrated to the appropriate oxygen atmosphere. CTLs were incubated with 10 μM EdU (ThermoFisher Scientific) at 37°C for 30 minutes to evaluate DNA synthesis in normoxia (18% O_2_) or hypoxia (1% O_2_). To terminate DNA synthesis, cells were fixed with 1% paraformaldehyde for 15 minutes at 22°C. Following fixation, cells were washed in 0.5% (v/v) FBS in PBS, and permeabilised with 0.5% (v/v) Triton X-100 in PBS for 15 minutes at 22°C. The incorporated EdU was labelled with Alexa 647-azide using a Click-IT chemistry reaction (ThermoFisher Scientific) at 22°C. After a 30 minute reaction, cells were washed twice using 0.5% (v/v) FBS in PBS. Cells were then stained with 50μg/ml propidium iodide in the presence of 50μg/ml ribonuclease A for 30 minutes at 22°C. Cells were resuspended in 0.5% (v/v) FBS in PBS for analysis. Data were acquired on a LSRFortessa with DIVA software (BD Biosciences). Data were analysed using FlowJo software (Tree Star).

### Antigen Receptor Triggering and ELISA Assays

Cells maintained in normoxia (18% O_2_) or hypoxia (1% O_2_) for 24 hours as described in “24-hour hypoxia treatments” were counted and resuspended in media (equilibrated to the appropriate gas environment overnight) at a concentration of 1×10^6^ cells per ml in RPMI 1640 with 10% FBS, 50 units/ml penicillin-G, 50 μg/ml streptomycin, 50 μM β-mercaptoethanol and 20 ng/ml IL-2. 1 ml of the cell suspension was plated per well of a 24 well plate. Cells were either mock stimulated with media, or stimulated with 100 ng/ml gp-33 peptide in the appropriate oxygen environment. After 4 hours, the supernatants from the wells were collected and subjected to ELISA analysis for IFNγ (IFN gamma ‘Femto-HS’ High Sensitivity Mouse Uncoated ELISA Kit, ThermoFisher Scientific), TNFα (TNF alpha Mouse ELISA Kit, ThermoFisher Scientific) and IL-10 (ELISA MAX™ Standard Set Mouse IL-10, BioLegend) according to the manufacturers’ protocols. Several serial dilutions of each sample were performed, to ensure that the sample detection was within the linear range of the ELISA kits. GraphPad Prism was used to fit standard curves, and determine the concentrations of IFNγ, TNFα and IL-10 in the samples.

### Statistical Analyses

Statistical analyses of changes in protein abundance from the proteomic data were performed as outlined in ‘Proteomic data processing and analysis’. Data derived from the proteomic studies (such as the mass of cells or mass of glycolytic enzymes per cell) and validation data were subjected to similar statistical analyses using a two-tailed paired Student’s t-test, without further adjustment.

## Results

### Hypoxia Reconfigures the CTL Proteome Selectively

**Figure 1:**
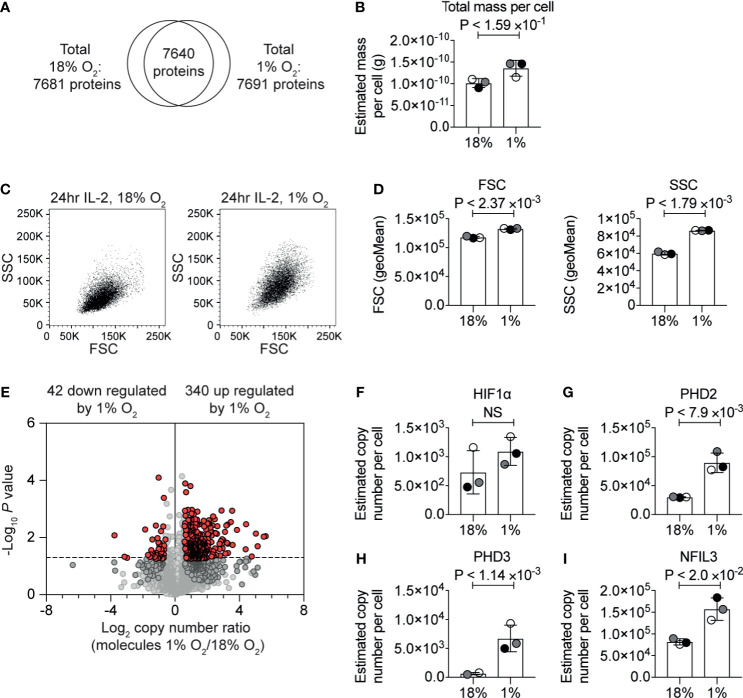
The impact of hypoxia on the CTL proteome. **(A)** Venn diagram showing the overlap of proteins identified in the proteomic analysis of IL−2 maintained CTLs in normoxia (18% O_2_) and IL−2 maintained CTLs subjected to hypoxia (1% O_2_) for 24 hours. **(B)** Protein content of CTLs in normoxia or subjected to hypoxia for 24 hours calculated using the proteomic data. **(C)** Representative flow cytometry FSC and SSC profiles for CTLs in normoxia or after 24 hours in hypoxia. **(D)** Bar charts showing the average of the geometric mean for the FSC and SSC profiles of three biological replicates of CTLs maintained in normoxia or subjected to hypoxia for 24 hours which were acquired at the same time. **(E)** Volcano plot showing the ratio of the protein copy number in CTLs in hypoxia for 24 hours compared to CTL maintained in normoxia, plotted against P value (two-tailed paired t-test). Proteins significantly changed (P ≤0.05) by greater than 1.5-fold are shown in red. **(F–I)** Copy numbers estimated from the proteomic data for the indicated proteins. Data show **(A, B, D–I)**, or are representative of, **(C)**, three biological replicates. In bar charts, data points from each of the three biological replicates are shown and are colour matched, the bar shows the mean and the error bars show standard deviation. P values were calculated using a two-tailed paired t-test. Non-significant changes are marked NS.

We generated a primary culture of CTLs from P14 TCR transgenic mice and then exposed a population to a gas atmosphere of 1% oxygen for 24 hours whilst maintaining a parallel population in standard culturing conditions with a gas atmosphere of 18% O_2_. In these experiments, CTLs were maintained in IL-2 to sustain viability and effector function. We then used quantitative high-resolution mass spectrometry to determine the proteomes of the CTLs maintained in normoxia (18% O_2_) *versus* hypoxia (1% O_2_). For each condition, over 7,600 protein groups were identified (**Figure 1A** and **Supplementary Figures S1A, B**, **Supplementary Table S1**), and there was >97% overlap in the proteins identified between biological replicates (**Supplementary Figures S1A, B**). Protein copies per cell were estimated using the ‘proteomic ruler’ method which uses the mass spectrometry signal of histones as an internal standard, providing normalised and quantitative data that is determined independently from the numbers of cells or total protein content of the sample (47).

The correlation between estimated copy numbers of proteins in different biological replicates of each condition was good, with the lowest R^2^-value being 0.91 (**Supplementary Figures S1C, D**). These data revealed that there was a slight increase in protein mass in the hypoxic T cells (**Figure 1B** and **Supplementary Table S2**). This correlated with flow cytometry analysis which indicated that CTL subjected to hypoxia had a slight increase in forward scatter (FSC), and a larger increase in side scatter (SSC) (**Figures 1C** **, D**). Using the protein copy number data, we calculated ratios of protein abundance of ~7,600 proteins that were identified in both normoxic and hypoxic CTLs (**Figure 1E** and **Supplementary Table S1**).

Consistent with cells being exposed to low oxygen levels, we detected an increase in HIF1α expression in CTL subjected to 1% oxygen (**Figure 1F**), which we have previously observed in CTLs (34). The increase in HIF1α expression was not found to be statistically significant. However, in accordance with activation of HIF1α-mediated transcription, we observed an increase in the abundance of the proteins encoded by HIF1 target genes notably the prolyl hydroxylases PHD2 (**Figure 1G**) and PHD3 (**Figure 1H**) and the transcription factor NFIL3 (**Figure 1I**).

In total, we identified 340 proteins that were significantly (P ≤0.05, two-tailed paired t-test) increased by >1.5-fold in response to hypoxia (**Supplementary Table S1**). The 20 proteins most upregulated in response to hypoxia included Interleukin-1 receptor type 2 (IL1R2, also known as CD121b) which acts as a decoy receptor for IL-1, which had <15-fold higher abundance in hypoxic cells than normoxic cells; the serine proteinase inhibitor, SERPINE1; and a predicted transmembrane protein, TMEM71. The top 20 proteins most increased in response to hypoxia also included NDRG1 which has been linked to driving T cell anergy (48).

Interestingly, only 42 proteins were significantly decreased >1.5-fold in CTL in response to hypoxia (**Supplementary Table S1**). Of these, the proteins that were most decreased in abundance in hypoxia included NDUFA4, which is a subunit of the cytochrome c oxidase which drives oxidative phosphorylation; fatty acid desaturase 1 (FADS1), succinate dehydrogenase cytochrome b560 subunit (SDHC); and the transcription factor eomesodermin (EOMES) (**Supplementary Table S1**).

### Impact of Hypoxia on CTL Metabolism

**Figure 2:**
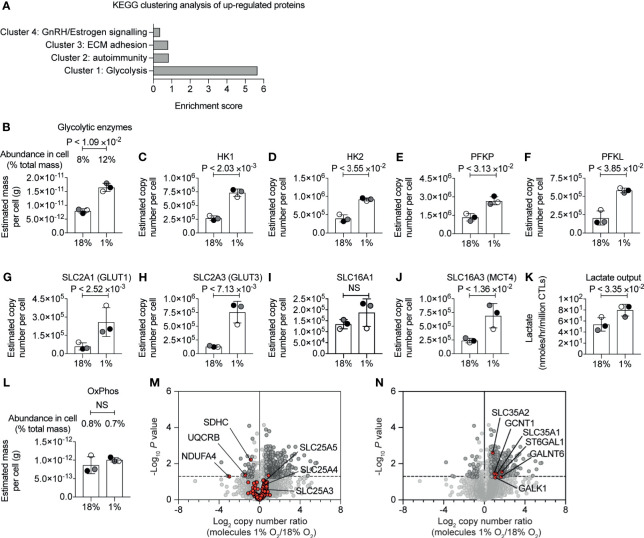
Hypoxia-mediated changes to proteins involved in glucose metabolism. **(A)** Proteins increased in abundance in CTLs after 24 hours in hypoxia were evaluated for function using DAVID. The bar chart shows the enrichment scores for the clusters. **(B)** Graph showing the estimated mass of proteins in a cell, calculated using the proteomic ruler, annotated with the KEGG pathway term “glycolysis”. **(C–J)** Copy numbers estimated from the proteomic data for the indicated proteins. **(K)** Lactate output in normoxic and hypoxic CTLs. **(L)** Bar chart showing the estimated mass of proteins in a cell, calculated using the proteomic ruler, annotated with the KEGG pathway term “Oxidative phosphorylation”. **(M, N)** Volcano plots showing the protein copy number ratio in hypoxic *vs* normoxic CTLs. In **(M)** mitochondrial proteins involved in oxidative phosphorylation and metabolite transport are shown in red, with selected proteins annotated. In **(N)** proteins increased in abundance in hypoxic CTLs and involved in cellular glycosylation are highlighted in red and are annotated. Data in **(A–N)** show three biological replicates. In bar charts, data points from the individual biological replicates are shown and are colour matched, the bar shows the mean and the error bars show standard deviation. P values were calculated using a two-tailed paired t-test. Non-significant changes are marked NS.

KEGG pathway clustering analysis revealed that the proteins increased in abundance in hypoxic CD8^+^ T cells were enriched in proteins involved in glycolytic metabolism (**Figure 2A** and **Supplementary Table S1**). Indeed, our proteomic data revealed the magnitude of this response. While approximately 8% of the mass of the P14 CTLs in normoxia comprised of glycolytic enzymes (**Figure 2B** and **Supplementary Table S2**), hypoxia triggered glycolytic enzymes to double in abundance in CTLs, increasing their dominance to ~12% of the CTL proteome (**Figure 2B** and **Supplementary Table S2**). This included substantial increases in rate-limiting enzymes for glycolysis (49), hexokinase 1 (HK1), hexokinase 2 (HK2) and phosphofructokinase (PFK) platelet and liver isoforms, which were expressed at 2-3 times higher levels in hypoxic CTL than normoxic CTL (**Figures 2C** **–F**). Indeed, all glycolytic enzymes increased in abundance in response to hypoxia (**Supplementary Figure S2**).

Hypoxia also increased the abundance of glucose and lactate transporters which are known to be rate limiting for glycolysis (49). CTLs express the glucose transporters SLC2A1 (also known as GLUT1) and SLC2A3 (also known as GLUT3) and the expression of both increased in response to oxygen deficiency (**Figures 2G** **, H**). SLC2A1 increased from ~60,000 copies per cell in normoxia to ~260,000 copies per cell in hypoxia, and SLC2A3 increased from ~130,000 copies per normoxic CTL to ~760,000 copies per hypoxic CTL. We identified the lactate transporters SLC16A1 (MCT1) and SLC16A3 (MCT4) in CTL. While there was a moderate impact of hypoxia on SLC16A1, SLC16A3 increased from ~240,000 copies per normoxic CTL to ~690,000 copies per hypoxic CTL (**Figures 2I** **, J**). These dramatic changes in expression of the rate limiting transporters for glycolysis suggested that CTLs in hypoxia might increase glycolytic metabolism. Accordingly, we measured lactate output to quantify glycolysis and found that there was an increase in lactate released into the extracellular environment from CTLs that had adapted to hypoxia compared to CTLs maintained in normoxia (**Figure 2K**).

The increase in abundance of glycolytic enzymes in hypoxic CTLs was not accompanied by substantial changes in the total mass of enzymes involved in oxidative phosphorylation (**Figure 2L** and **Supplementary Table S2**). However, we did detect decreases in specific components of the mitochondrial oxidative phosphorylation machinery. This included succinate dehydrogenase cytochrome b560 subunit (SDHC), belonging to complex II  (50), which decreased in abundance ~2 fold in hypoxic cells (**Figure 2M**); ubiquinol-cytochrome c reductase binding protein (UQCRB), which is part of complex III (51), also decreased in abundance ~2 fold in response to hypoxia; and NDUFA4, which was thought to belong to complex I, but is now thought to be important for the function of complex IV (52), decreased ~10-fold in response to hypoxia. Consequently, although there is not an overall decrease in the total mass of enzymes associated with mitochondria, there could be a change in oxygen consumption by cells in response to hypoxia as a consequence of loss of these specific components.

There were no changes in expression of mitochondrial transport proteins that are responsible for transporting metabolites between the cytosol and mitochondria (**Figure 2M** and **Supplementary Table S1**). This includes the highly abundant SLC25A4 and SLC25A5, that transport ADP from the cytoplasm into the mitochondrial matrix and ATP from the mitochondrial matrix into the cytoplasm, and SLC25A3, which transports phosphate into mitochondria.

We also assessed if hypoxia induced a change in the abundance of proteins that control other glucose metabolic pathways. We found that hypoxia had no impact on expression of the key enzymes that mediate the TCA cycle, but we did observe modest changes in expression of proteins of the pentose phosphate pathway (PPP) namely 6-phosphogluconate dehydrogenase and Transketolase (**Supplementary Figure S3**). We also observed that hypoxia triggered an increase in expression of SLC35A1 and SLC35A2 (**Figure 2N**), transporters that deliver nucleotide-sugars into the Golgi apparatus or Golgi vesicles. In addition, several proteins that control protein glycosylation were increased in abundance in hypoxic T cells including polypeptide N-acetylgalactosaminyltransferase 6 and beta-galactoside alpha-2,6-sialyltransferase 1 (**Figure 2N**). We also noted that hypoxia increased abundance of isocitrate dehydrogenase (IDH1), which is involved in the metabolism of glutamine, ATP-citrate synthase (ACLY), which metabolises citrate, and acetyl-coenzyme A synthetase (ACSS2), which metabolises acetate (**Supplementary Figure S2**).

### Impact of Hypoxia on Protein Biosynthesis Machinery

**Figure 3:**
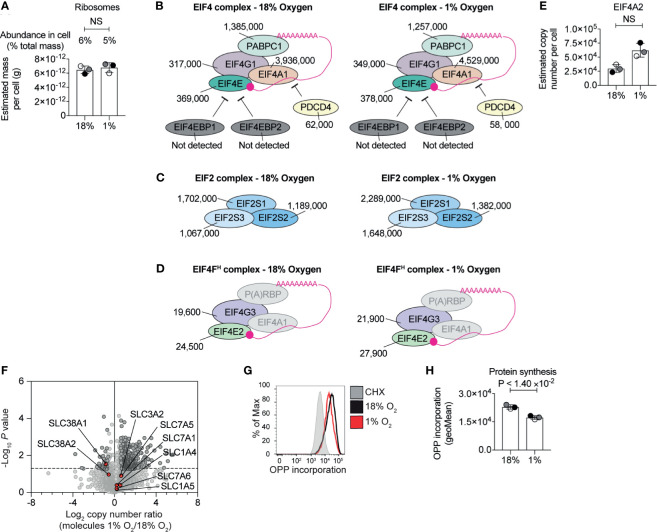
Regulation of protein synthesis machinery by hypoxia. **(A)** Graph showing the estimated mass of proteins in a cell, calculated using the proteomic ruler, annotated with the KEGG pathway term “ribosome” for CTLs maintained in normoxia (18% O_2_) or subjected to 24 hours of hypoxia (1% O_2_). **(B–D)** Schematic representation of the EIF4 complex **(B)** and EIF2 complex **(C)** and the EIF4F^H^ complex **(D)**. P(A)RBP stands for poly(A) RNA binding proteins. Average copy numbers for three biological replicates of CTLs maintained in normoxia or subjected to 24 hours of hypoxia are annotated alongside. **(E)** Copy numbers estimated from the proteomic data for EIF4A2. **(F)** Volcano plot showing the protein copy number ratio in hypoxic *vs* normoxic CTLs with amino acid transporters highlighted in red and annotated. **(G, H)** Protein synthesis of CTLs maintained in normoxia or subjected to 24 hours of hypoxia was evaluated using the puromycin analogue, OPP using a flow-cytometry-based assay. Normoxic CTLs were pre-treated with cycloheximide (CHX) for 30 minutes to block translation before the addition of OPP. Representative flow cytometry plots of one biological replicate of the OPP incorporation are shown in **(G)** and the geometric mean of the fluorescence intensity for all three biological replicates acquired at the same time are shown in **(H)**. Data in **(A)**, **(E, F)** show three biological replicates; data in **(B–D)** show average values from three biological replicates. In bar charts, data points from biological replicates are shown and are colour matched, the bar shows the mean and the error bars show standard deviation. P values were calculated using a two-tailed paired t-test. Non-significant changes are marked NS.

Changes to the proteome depend on transcription and translation. The substantial increase in the highly abundant glycolytic enzymes are consistent with substantial levels of protein synthesis. Yet, it has been reported that severe hypoxia limits protein translation in cells to conserve energy (43, 53, 54). CTLs are known to express high levels of ribosomes, tRNA synthetases, eukaryotic initiation factor 4 (eIF4) complexes that translate methyl capped mRNAs and EIF2 complexes which control tRNA transfer to ribosomes (5). Our experiments revealed no substantial loss of ribosome mass (**Figure 3A** and **Supplementary Table S2**) or any of these key translational complexes in hypoxic T cells (**Figures 3B** **, C**). We did not detect any up-regulation of translational repressors such as 4E-BP1 and 4E-BP2 (55), which bind to eIF4E to prevent the assembly of translational initiation complexes, or PDCD4, which inhibits translation by binding to EIF4A1 (56) (**Figure 3B**). We identified EIF4E2 and EIF4G3 (**Figure 3D**) components of the EIF4F^H^ mRNA cap-binding complex activated during hypoxia and several poly(A) RNA binding proteins (**Supplementary Table S1**) that have been linked to reprogramming the translatome of cells to allows cells to adapt to low oxygen (43). None of these proteins appear to increase in response to hypoxia, and the abundance of the EIF4F^H^ is relatively low compared to the basal EIF4F complex (comprising of EIF4E1 and EIF4G1). Interestingly, our data indicated there was an increase in EIF4A2 (**Figure 3E**), a paralog of EIF4A1, which can act as a translational repressor in some contexts (57). Moreover, hypoxic T cells retained high levels of expression of critical amino acid transporters, such as the system L transporter SLC7A5, (**Figure 3F**) that fuels protein synthesis in CTL (58, 59). These data suggest that overall, CTLs experiencing hypoxia retain the cellular machinery required to carry out protein synthesis, and do not upregulate proteins that are known to reprogramme translation in response to hypoxia.

In order to assess the activity, rather than the abundance, of the protein synthesis machinery in hypoxic CTL, we used a quantitative single-cell assay that measures protein synthesis by detecting the catalytic incorporation of O-propargyl-puromycin (OPP), an analogue of puromycin and aminoacyl-tRNA mimetic, into elongating nascent protein chains in the ribosome. These experiments determined that CTLs in normoxia and hypoxia incorporated high levels of OPP. There was a small reduction in OPP incorporated by hypoxic CTL compared to normoxic cells (**Figures 3G** **, H**), suggesting that while the abundance of the translational machinery was not impacted by hypoxia, there may be an impact on its activity. However, this was not a substantial effect. Indeed, the increased abundance of highly abundant cellular glycolytic enzymes and the overall increase in cell mass and expression of more than 300 proteins in CTL cultured in 1% O_2_ is consistent with hypoxic CTL retaining relatively high protein synthetic capacity, as well as the preferential translation of genes required for the adaptation to hypoxia.

### Hypoxia Limits the Proliferation of IL-2 Maintained CTLs

**Figure 4:**
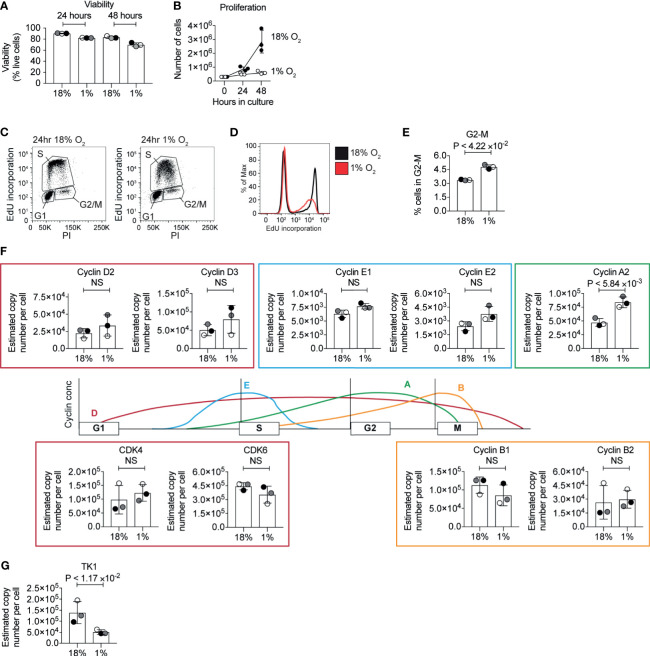
Inhibition of the cell cycle by hypoxia. **(A)** Viability of CTL maintained in normoxia or subjected to hypoxia for 24 hours or 48 hours as determined by flow cytometry forward scatter (FSC) and side scatter (SSC) and DAPI staining. **(B)** Proliferation of CTLs in normoxia or hypoxia over 48 hours. **(C–E)** Flow cytometric analysis of cell cycle stages in CTLs maintained in normoxia or after 24 hours of hypoxia using EdU incorporation into DNA. Representative flow plots of DNA stained with PI against EdU incorporation are shown in **(C)**, and a histogram of EdU incorporation alone is shown in **(D)**. The percentage of CTL identified as in G2 is shown in **(E)**. **(F)** Schematic representation of selected cyclin levels during the cell cycle, with bar charts showing copy numbers estimated from the proteomic data for the indicated cyclins and CDKs shown alongside. **(G)** Copy numbers for thymidine kinases estimated from the proteomic data. Data in **(A, B)** and **(E–G)** show three biological replicates. Data in **(C, D)** are representative of three biological replicates. In bar charts, data points from individual biological replicates are shown and are colour matched, the bar shows the mean and the error bars show standard deviation. P values were calculated using a two-tailed paired t-test. Non-significant changes are marked NS.

Cell cycle arrest and inhibited proliferation is triggered in many cell types in response to hypoxia (60). In T cells, low oxygen has been reported to inhibit (18, 22–25) or increase (61, 62) CD8^+^ T cell proliferation in different contexts. CTLs cultured in normoxia clonally expand when maintained in exogenous IL-2. We, therefore, investigated the proliferation of CTLs that were generated by activating naïve P14 CD8^+^ T cells with peptide antigen for 48 hours before differentiation in IL-2 for 3 days in normoxia. These cells were then either maintained in IL-2 in normoxia or maintained in IL-2 and subjected to hypoxia, with cell counts being taken 24 and 48 hours after cells were first exposed to hypoxia. Cell counting revealed that the CTLs in hypoxia survive, but do not proliferate (**Figures 4A** **, B**). To determine if hypoxia caused T cells to arrest at any particular stage of the cell cycle, we assessed the DNA content of the cells using the DNA-binding dye propidium iodide and evaluated DNA synthesis by measuring the incorporation of EdU into DNA. We detected both of these parameters by flow cytometry. Hypoxic CTLs showed decreased EdU incorporation compared to control CTLs in 18% O_2_ (**Figures 4C** **, D**). These data reveal that CTLs experiencing hypoxia are impaired in their ability to progress through S phase and hence fail to proliferate. It has been proposed that PHD1-mediated prolyl hydroxylation of the centrosomal protein Cep192 regulates its proteasomal degradation, and its abundance on the centrosome to control the formation of the mitotic spindle (38). We found that the frequency of hypoxic CTLs in the G2-M phase of the cell cycle is ~5% which is ~1.5 fold of the frequency of that measured in normoxic T cells (**Figure 4E**). This could indicate some delay in mitosis in hypoxic CTLs, however, the cell cycle phenotype was not consistent with a full mitotic block. Moreover, we did not detect PHD1 nor Cep192 in CTLs. Thus, changes to DNA synthesis appear to be the major driver of the changes in proliferation of hypoxic CTLs.

In cancer cells, changes in DNA replication have been associated with decreased levels of MCM proteins, and inhibited origin firing, caused by an interaction between HIF1 and CDC6, which loads the MCM helicase complex onto DNA (60). We interrogated the proteomic data to determine whether hypoxia altered the abundance of key cell cycle regulatory proteins. We did not detect changes in the abundance of MCM proteins in our proteomics analysis. Normoxic and hypoxic T cells expressed similar levels of the D2 type cyclins, Cyclins D2 and D3, and the associated cyclin dependent kinases CDK4 and CDK6, but CTLs in hypoxia had increased expression of cyclin A2 (**Figure 4F**). However, hypoxic CTLs showed decreased abundance of thymidine kinase 1 (**Figure 4G**) which synthesises thymidine monophosphate from thymidine, a crucial phosphorylation step that is required to allow thymidine to be incorporated into DNA (63). Therefore, this decrease in TK1 could explain the decreased DNA synthesis rates seen in hypoxic CTLs. However, as the abundance of TK1 and cyclins are regulated by the cell cycle, it is unclear whether changes in expression of these proteins is a cause, or a consequence, of changes to the altered cell cycle progression of hypoxic CTLs.

### Hypoxia Controls Expression of CTL Effector Molecules

**Figure 5:**
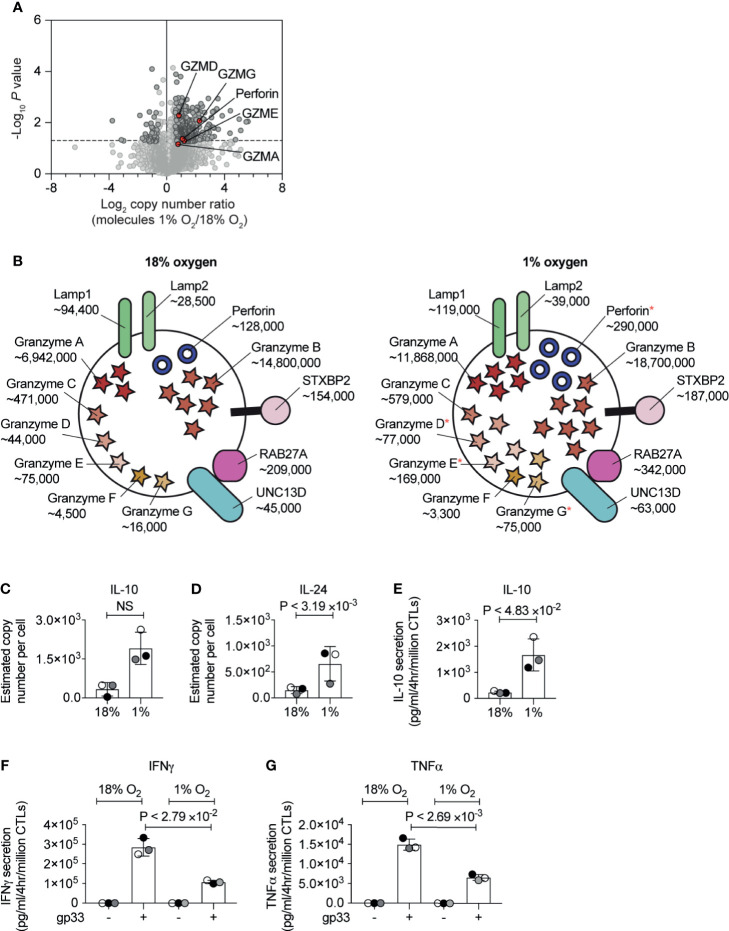
The consequences of hypoxia on proteins involved in CTL cytolytic function. **(A)** Volcano plot showing the protein copy number ratio in CTLs subjected to 24 hours of hypoxia (1% O_2_) compared to CTLs maintained in normoxia (18% O_2_) with granzymes and perforin highlighted in red and annotated. **(B)** Schematic representation of secretory lysosomes showing cytolytic molecules and proteins required for degranulation. Average copy numbers for three biological replicates of CTLs maintained in normoxia or subjected to 24 hours of hypoxia are annotated alongside, with * indicating the proteins that increase significantly in abundance in hypoxia. **(C, D)** Copy numbers estimated from the proteomic data for IL-10 and IL-24. **(E–G)** CTLs were maintained in normoxia or subjected to hypoxia for 24 hours. Cytokine released by the CTLs was measured over a 4 hour period in normoxia or hypoxia. In **(E)** IL-10 was measured in unstimulated CTLs, and IFNγ **(F)** and TNFα **(G)** were measured in unstimulated cells and cells stimulated with the gp33 peptide. Data in **(A, C–G)** show three biological replicates. Data in **(B)** show average values from three biological replicates. In bar charts, data points from each biological replicates are shown and colour matched, the bar shows the mean and the error bars show standard deviation. P values were calculated using a two-tailed paired t-test. Non-significant changes are marked NS.

Previous studies have shown that in both normoxia and hypoxia, the expression of the CTL effector molecule, perforin is controlled by HIF1-dependent signalling pathways mediated by the transcription factor NFIL3 (34). Our proteomic data confirmed an increase in the abundance of perforin in hypoxic CTL (**Figure 5A**), and also showed that hypoxic CTLs increase the abundance of granzyme A, as well as the lower abundance granzyme D, granzyme E and granzyme G (**Figure 5A**). Perforin and granzymes are stored within specialised secretory lysosomes marked by the lysosomal proteins LAMP1 (also known as CD107a) and LAMP2. There was no substantial increase in LAMP1 or LAMP2 expression in hypoxic CTLs (**Figure 5B**). Moreover, expression of the GTPase RAB27A and STXBP2 (MUNC18-2), UNC13D (MUNC13-4) which control the regulated secretion of T cell cytolytic granules did not substantially, or reproducibly, change in abundance in hypoxic CTL (**Figure 5B**). These data reveal that hypoxia did not generally increase the quantity of secretory machinery or secretory lysosomes in CTL, but rather stimulated the loading of granules with effector molecules, consistent with previous studies (23).

Another effector function of CTL is to secrete cytokines and the current experiments detected IL-10, IL-24, TNF and LTA in both normoxic and hypoxic CTL (**Supplementary Table S1**). There were decreases in the expression of TNF and LTA in response to hypoxia but more striking was the increased expression of the anti-inflammatory cytokine IL-10 (**Figure 5C**) and IL-24 (**Figure 5D**), another IL-10 family member. Previously IL-10 has been shown to increase in hypoxic CTLs in response to TCR triggering (22), but also in unchallenged hypoxic CTLs (23). To validate that CTL in hypoxia secreted more IL-10 in the absence of exogenous antigen, we used an ELISA assay. We found that CTL in normoxia only secreted low levels of IL-10, however, CTL adapted to hypoxia showed increased production of IL-10 (**Figure 5E**).

IFN*γ* is an important pro-inflammatory cytokine produced by CTLs during an immune response. We did not detect intracellular IFN*γ* in CTL cultured in normoxia or hypoxia in our proteomic dataset. This may reflect that this cytokine is immediately secreted from CTLs and is not stored in any intracellular compartment. Therefore, we used ELISAs to examine the impact of hypoxia on basal and antigen receptor induced production of IFN*γ* by CTLs. We found that hypoxia did not impair the low basal IFN*γ* production by CTLs (**Figure 5F**). However, antigen-stimulated IFN*γ* was decreased in CTLs that had adapted to hypoxia for 24 hours (**Figure 5F**). Moreover, we detected a similar reduction of TNFα production in hypoxic CTLs compared to normoxic CTLs (**Figure 5G**).

### Hypoxia Regulation of T Cell Membrane Proteins

**Figure 6:**
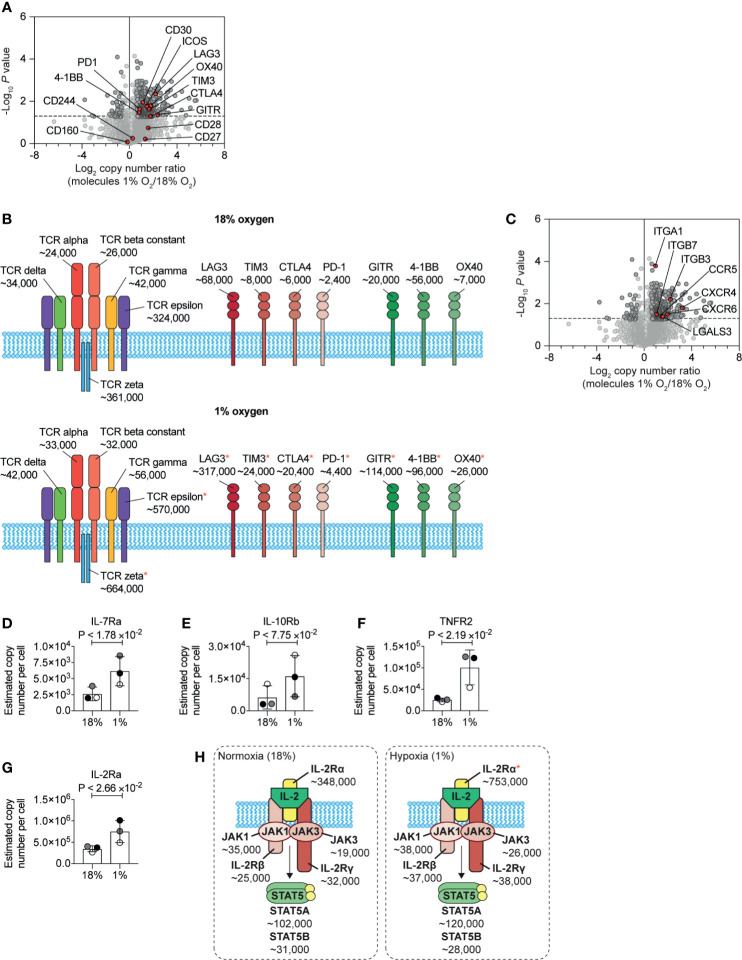
Changes to plasma membrane receptors induced by hypoxia. **(A)** Volcano plot showing the protein copy number ratio in CTLs subjected to 24 hours of hypoxia (1% O_2_) compared to CTLs maintained in normoxia (18% O_2_) with co-stimulatory and inhibitory checkpoint molecules annotated and highlighted in red. **(B)** Schematic representation of the T cell receptor (TCR) and selected co-stimulatory and inhibitory checkpoint molecules. Average copy numbers for three biological replicates of CTLs maintained in normoxia or subjected to 24 hours of hypoxia are annotated alongside, with * indicating the proteins that increase significantly in abundance in hypoxia. **(C)** Volcano plot showing the protein copy number ratio in CTLs subjected to 24 hours of hypoxia (1% O_2_) compared to CTLs maintained in normoxia (18% O_2_) with selected adhesion molecules and chemokine receptors highlighted in red. **(D–G)** Copy numbers estimated from the proteomic data for the indicated proteins. **(H)** Schematic representation of the IL-2 receptor and JAK-STAT5 signalling pathway. Average copy numbers for three biological replicates of CTLs maintained in normoxia or subjected to 24 hours of hypoxia are annotated alongside. Data in **(A, C–G)** show three biological replicates. Data in **(B, H)** show average values from three biological replicates. In bar charts, data points from each biological replicate are shown, and are colour matched, the bar shows the mean and the error bars show standard deviation. P values were calculated using a two-tailed paired t-test.

Previous flow cytometry studies have shown that HIF mediated signalling pathways control the expression of the costimulatory molecules CD137, OX40, and GITR, and the inhibitory checkpoint receptors PD-1, TIM3, and LAG3 (26). In our unbiased proteomic analyses, we identified that hypoxia upregulated multiple stimulatory checkpoint receptors, including ICOS, GITR, 4-1BB, and OX40, and inhibitory receptors PD-1, CTLA-4, Tim-3 and Lag-3 (**Figure 6A**). In contrast, expression of the T cell antigen receptor complex (**Figure 6B**) and other costimulatory molecules such as CD160 and CD244 (2B4) (**Figure 6A**) remained relatively unchanged. However, we did detect an increase in the abundance of the CD3ϵ (T-cell surface glycoprotein CD3 epsilon chain) and CD3ζ (T-cell surface glycoprotein CD3 zeta chain) components of the T cell receptor (**Figure 6B**). Within hypoxic tumours, elevated levels of adenosine have been shown to be an important metabolic checkpoint by which T cells are inhibited (64, 65). However, we did not identify the A2A adenosine receptor nor the A2B adenosine receptor in our proteomic dataset.

One new insight from this quantitative proteomic data, that cannot be determined from flow cytometry or mRNA data, is the differential abundance of the different costimulatory and inhibitory checkpoint receptors on CTLs. Normoxic CTLs express approximately ~7×10^4^, ~8×10^3^ and ~6×10^3^ copies of the inhibitory checkpoint molecules LAG3, TIM3 and CTLA4 respectively, with PD-1 being the least abundant at ~2.5×10^3^ molecules per cell. In addition, under normoxia, CTLs expressed ~6×10^4^, ~2×10^4^ and ~7×10^3^ molecules of the costimulatory molecules 4-1BB, GITR and OX40 respectively (**Figure 6B**). In response to hypoxia, LAG-3 increases ~4.5-fold to ~3×10^5^ copies per cell, becoming the most abundant inhibitory or stimulatory checkpoint receptor expressed by CTLs (**Figure 6B**). Interestingly, as GITR increased ~5-fold, compared to the ~1.5-fold induction of 4-1BB, these molecules were subsequently expressed at a similar level of ~1×10^5^ copies per cell (**Figure 6B**). These data, therefore, reveal how hypoxia can alter the dominance of these molecules in CTLs.

Additionally, hypoxia caused CTLs to increase the abundance of multiple molecules involved in cell adhesion and trafficking including integrins, galectin-3 and many chemokine receptors including CCR5, CXCR6 and CXCR4 (**Figure 6C**). These latter chemokine receptors are known to control T cell trafficking into tumours and sites of inflammation. These changes support a model that hypoxia will promote T cell recruitment and retention to sites of inflammation rather than localisation in lymphoid tissues. However, we found that hypoxia increased the abundance of CD62L in CTLs (**Supplementary Table S1**). Previous studies have shown that activation of HIF transcriptional programs repress expression of the adhesion molecule, CD62L (28) and repress the ability of CTL to traffic into secondary lymphoid tissues (26, 28). It is, therefore, possible that an increase in CD62L in response to hypoxia may be triggered by pathways other than increased transcription, such as inhibited shedding of the ectodomain (66). Indeed, as elevated CD62L has been associated with increased ability of CD8^+^ T cells to infiltrate sites of infection and tumours (67, 68), it is possible that hypoxia-induced upregulation of CD62L may also be beneficial for allowing CD8^+^ T cells to enter diseased tissues.

We also identified that hypoxia increased the abundance of a number of cytokine receptors expressed by CTLs, which may then confer sensitivity to different immune stimuli in their environment. We observed an increase in the IL-7 receptor alpha chain (IL-7Ra) (**Figure 6D**). Interestingly the beta subunit of the IL-10 receptor (IL10RB) was reproducibly increased in abundance in hypoxic CTLs, although this increase was not statistically significant (**Figure 6E**), Notably, the tumour necrosis factor receptor superfamily member 1B (TNFRSF1B also known as tumour necrosis factor receptor 2, TNFR2) increased from ~25,000 copies per cell in normoxia to ~100,000 copies per cell in hypoxia (**Figure 6F**). Hypoxia also caused CTLs to increase the abundance of the IL-2 receptor alpha chain (IL-2Ra) (**Figures 6G, H**). However, the rate limiting components of the IL-2 receptor complex, the IL-2R beta chain and the common gamma chain did not show substantial or reproducible changes in abundance in response to hypoxia. Nor were there any changes in expression of the tyrosine kinases JAK1 and JAK3, that mediate signalling by the IL-2 receptor (**Figure 6H**). Therefore, the impact of hypoxia on the expression of cytokine receptors, was also highly selective.

## Discussion

**Figure 7:**
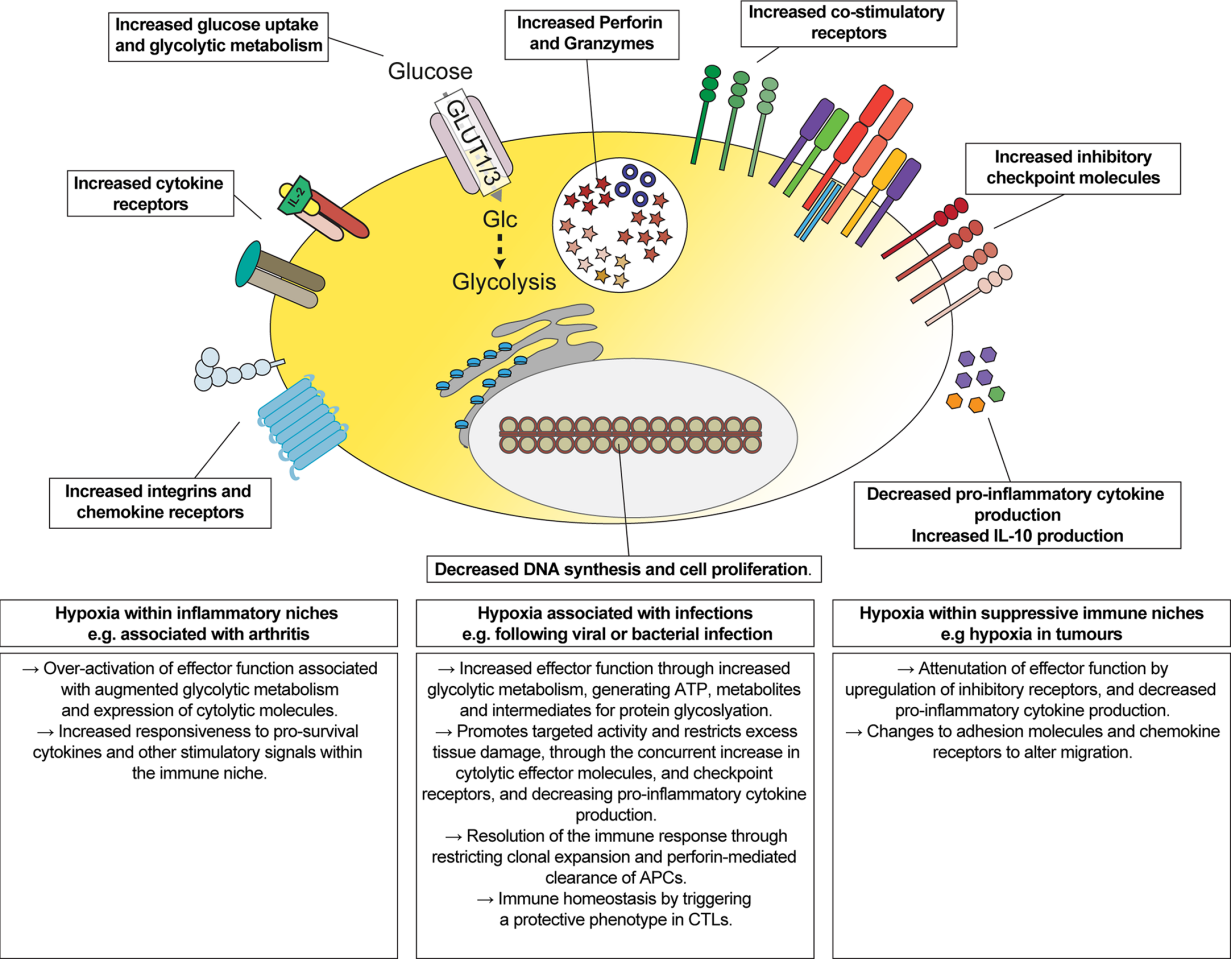
Overview of the impact of hypoxia on CTLs. Schematic representation of changes induced by hypoxia on CTLs, and their implications for regulating the function of CTLs in different immune environments.

The impact of hypoxia, and activation of oxygen-sensing signalling pathways, has been reported to both stimulate (23, 26–28, 32, 33) and inhibit (69) CD8^+^ T cell functions. Here, we provide a quantitative and comprehensive understanding of the impact of hypoxia on the proteome of differentiated CTLs. Our data highlight that hypoxic environments may stimulate some functions of CTL, such as increasing the abundance of cytolytic molecules, but these changes are accompanied by a parallel increase in the abundance of inhibitory checkpoint molecules and the cytokine IL-10, and a decreased ability to produce pro-inflammatory cytokines in response to antigen-receptor stimulation (**Figure 7**).

These data are consistent with oxygen-dependent signalling pathways targeting the activity of CTLs towards cell-mediated killing whilst restricting excess tissue damage. Inhibitory receptors such as CTLA-4, PD-1 and LAG3 are not expressed by naïve T cells but are upregulated in activated T cells (70–72). The higher abundances of inhibitory checkpoint molecules, as well as the upregulation of perforin and granzymes, is consistent with hypoxic CTLs having an augmented cytolytic phenotype but where the cells may be limited in their ability to attack healthy cells. However, it is also possible that hypoxia may convert CTLs to having a regulatory phenotype. For example, IL-10-producing CD8^+^ effector T cells are associated with being protective during an immune response (73, 74). Indeed, subsets of CD8^+^ Tregs are characterised by the expression of markers including CD25, CTLA-4 or PD-1 (75–78). Moreover, an increase in IL-10 could further restrict the activity of cytolytic CTLs as IL-10 has been shown to decrease their sensitivity to antigens (79).

A major novelty, and advantage, of our quantitative proteomic data is that it provides information on the relative abundance of different proteins that cannot be inferred from gene transcripts, nor quantified using antibodies *via* flow cytometry or western blot. Therefore, although both co-stimulatory and inhibitory checkpoint molecules are upregulated in response to hypoxia, our data revealed that LAG3 was the most abundant inhibitory checkpoint molecule, and its substantial upregulation in response to hypoxia increased its abundance greater than that of GITR and 4-1BB combined. These changes in response to hypoxia could, therefore, shift the balance of CTLs towards responding to inhibitory signals within an immune environment. Consequently, the overall impact of activation of hypoxia-induced signalling pathways in CD8^+^ T cells may be dependent on the context, and other factors which can regulate the activity of the T cells in the immune environment, including immune and metabolic checkpoint molecules.

Another important and novel observation from this study was that hypoxia can override IL-2-induced DNA-synthesis and proliferation programmes. In the context of immune cells, it is an intriguing possibility that oxygen levels within an immune niche may play a role in limiting excessive proliferation. Additionally, up-regulation of perforin in CTL may be an important negative feedback control of their functions in hypoxic environments. Mutations that cause perforin deficiency in humans are associated with the disorder, Hemophagocytic lymphohistiocytosis (HLH) (80). Rather than causing immunodeficiency, loss of perforin causes systemic inflammation during immune activation, often triggered by infection (80). This inflammation, associated with excessive activation of CTLs and macrophages and a cytokine storm, is thought to be caused by the failure of CD8^+^ T cells to kill antigen-presenting dendritic cells (81). Therefore, our data reinforce a role for hypoxia, and oxygen-sensing signalling pathways, in controlling the balanced activity of CTLs, limiting their clonal expansion and promoting activities that may contribute to resolving an immune response.

Transcriptional programmes induced by HIF are likely to play a major role in controlling gene expression and functional changes in CTLs in response to hypoxia. Previous studies have shown that activation of HIF1-induced transcriptional pathways *via* knockout of PHD1/2/3 or VHL can augment the killing activities of CTLs, stimulating glycolytic metabolism and the expression of granzyme B and perforin and co-stimulatory molecules (27, 32, 33). Our data are consistent with these findings. Approximately 70 proteins increased in abundance in CTL in response to hypoxia were decreased at the transcript level in HIF1β-deficient CTLs: this included the transporters GLUT1, GLUT3 and SLC16A3; glycolytic enzymes; perforin; granzymes C, D, E and G; 4-1BB and ICOS; the IL-7 receptor alpha chain; IL-10; IL-24; Tim-3 and PD-1; chemokine and adhesion molecules; and the glycosylases ST6GAL1, GCNT1 and GALNT6 (28). Even so, some of these changes in mRNA or protein abundance could be indirect from HIF-mediated transcription. One important transcription factor in immune cells is NFIL3, and its expression in CD8^+^ T cells is HIF-dependent (28, 34). Interestingly, several proteins that increase in hypoxia have been reported to depend on NFIL3 in T cells. This includes Tim-3 (82), IL-10 (82, 83) and perforin (34). Consequently, these data indicate that some changes in gene expression that take place in hypoxic T cells may depend on NFIL3.

Approximately 30% of the proteins increased in abundance in hypoxia did not map to genes found to change in abundance in the microarray analysis of HIF1β knockout CTLs. While this could reflect differences in normalisation and statistical approaches that reveal genes or proteins that are regulated, it is also possible that up-to 270 proteins may be increased in CTLs in response to hypoxia *via* HIF-independent mechanisms. This could reflect regulation of protein stability through PHD family members (39) or the ADO-controlled N-degron pathway (44). Alternatively, changes to glucose metabolism may contribute to differential expression of proteins in response to hypoxia. For example, another novel observation from our study is that several proteins involved in protein glycosylations were increased in response to hypoxia. Therefore, these changes may alter the abundance of post-translational glycoslyations that control protein stability. Additionally, changes to mitochondrial respiration during hypoxia may be an important factor that influences gene expression. We identified components of mitochondrial complexes that were decreased in abundance in hypoxia, which have the potential to block mitochondrial-dependent signalling pathways. In CD4^+^ T cells, IFNγ production has been shown to be inhibited by loss of mitochondrial respiration (84). Therefore, these changes could be a factor which contributes to the decrease in IFNγ production in response to antigen stimulation in hypoxic CTLs compared to their normoxic counterparts.

This study provides an important resource quantifying changes in the abundance of proteins that are relevant for understanding how CTLs may function in hypoxic immune environments (**Figure 7**). Our data demonstrate how hypoxia may integrate with, and alter, cytokine and antigen-induced gene expression programmes. Recently it has been shown that chronic antigen stimulation in combination with hypoxia may promote T cell exhaustion *via* exacerbating mitochondrial dysfunction in tumour microenvironments (17, 85, 86). Although deficiency of VHL and PHD1/2/3 may not fully mimic hypoxia, it is also interesting to note that constitutive activation of the HIF-transcriptional pathway alone may also have context-specific phenotypes. For example, cell-intrinsic immunopathology was only observed in VHL-deficient CD8^+^ T cells in response to persistent antigen resulting from chronic infection, but not in response to acute infections or antigens associated with tumours, where constitutive HIF expression was beneficial (27). As hypoxia is a feature of immune responses in which pathogens can be cleared from the body and diseases which are exacerbated by immune cell dysfunction, it will be important to know more about the signals that may alter the balance of oxygen-sensing pathways within a hypoxic immune environment.

## Data Availability Statement

The original contributions presented in the study are publicly available. This data can be found here: https://www.ebi.ac.uk/pride/, PXD026223.

## Ethics Statement

The animal study was reviewed and approved by The Welfare and Ethical Use of Animals Committee (WEC), University of Dundee, Dundee, United Kingdom.

## Author Contributions

SR designed the project, designed and performed most experiments, and analysed the data. CR performed and analysed the CTL proliferation assay with input from SR. SR and DC wrote the manuscript with input from CR. All authors contributed to the article and approved the submitted version.

## Funding

This work was supported by the Wellcome Trust (Principal Research Fellowship 205023/Z/16/Z to DC) and Tenovus Scotland (SR). CR was the recipient of a studentship from the Biotechnology and Biological Sciences Research Council and GlaxoSmithKline. Additionally, this work was supported by the Biotechnology and Biological Sciences Research Council through Institute Strategic Program Grant funding, BBS/E/B/000C0427 and BBS/E/B/000C0428.

## Conflict of Interest

The authors declare that the research was conducted in the absence of any commercial or financial relationships that could be construed as a potential conflict of interest.

## Publisher’s Note

All claims expressed in this article are solely those of the authors and do not necessarily represent those of their affiliated organizations, or those of the publisher, the editors and the reviewers. Any product that may be evaluated in this article, or claim that may be made by its manufacturer, is not guaranteed or endorsed by the publisher.
