# Supplementary material for: Quantitative Analyses Reveal How Hypoxia Reconfigures the Proteome of Primary Cytotoxic T Lymphocytes
Source: Front Immunol. 2021 Sep 17;12:712402. doi: 10.3389/fimmu.2021.712402 (PMC8484760; doi:10.3389/fimmu.2021.712402)
Supplement: Supplementary file 1 [file DataSheet_1.pdf]

Figure S1

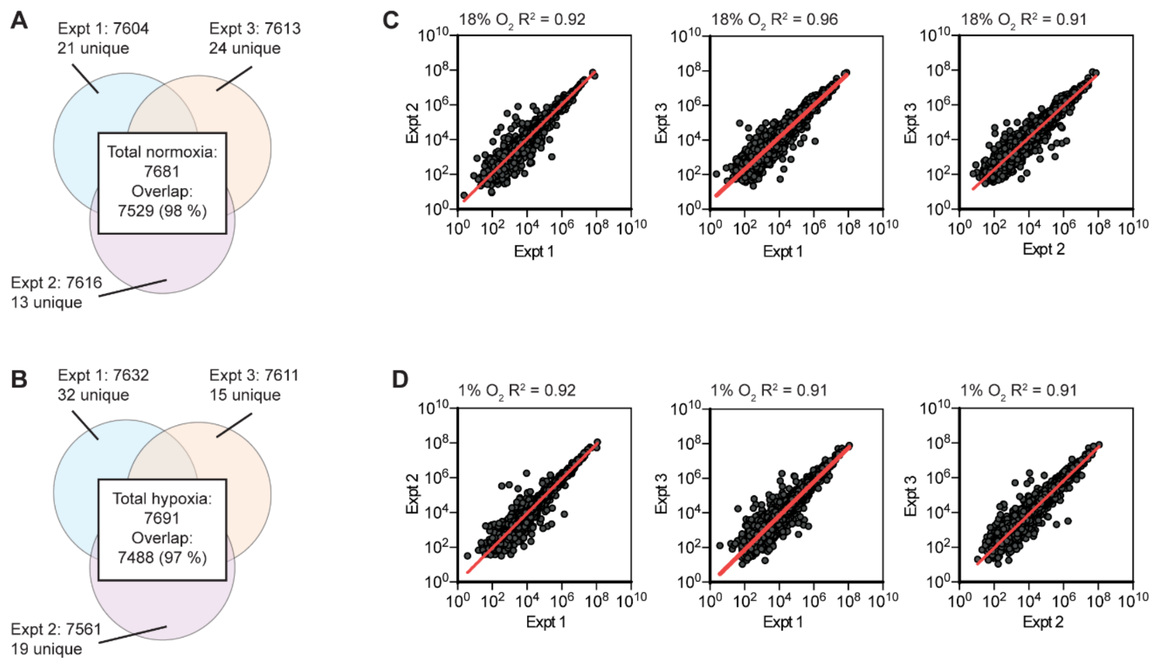

Figure S1: Overlap and correlation between protein identifications and quantifications in each biological replicate.

Overlap in protein identifications between the three biological replicates of proteomic analyses on CTL maintained in normoxia (18% O_2) (**A**) and subjected to 24 hours of hypoxia (1% O_2) (**B**). Correlation between copy numbers quantified for proteins in each biological replicate in normoxia (**C**) and hypoxia (**D**).

Figure S2

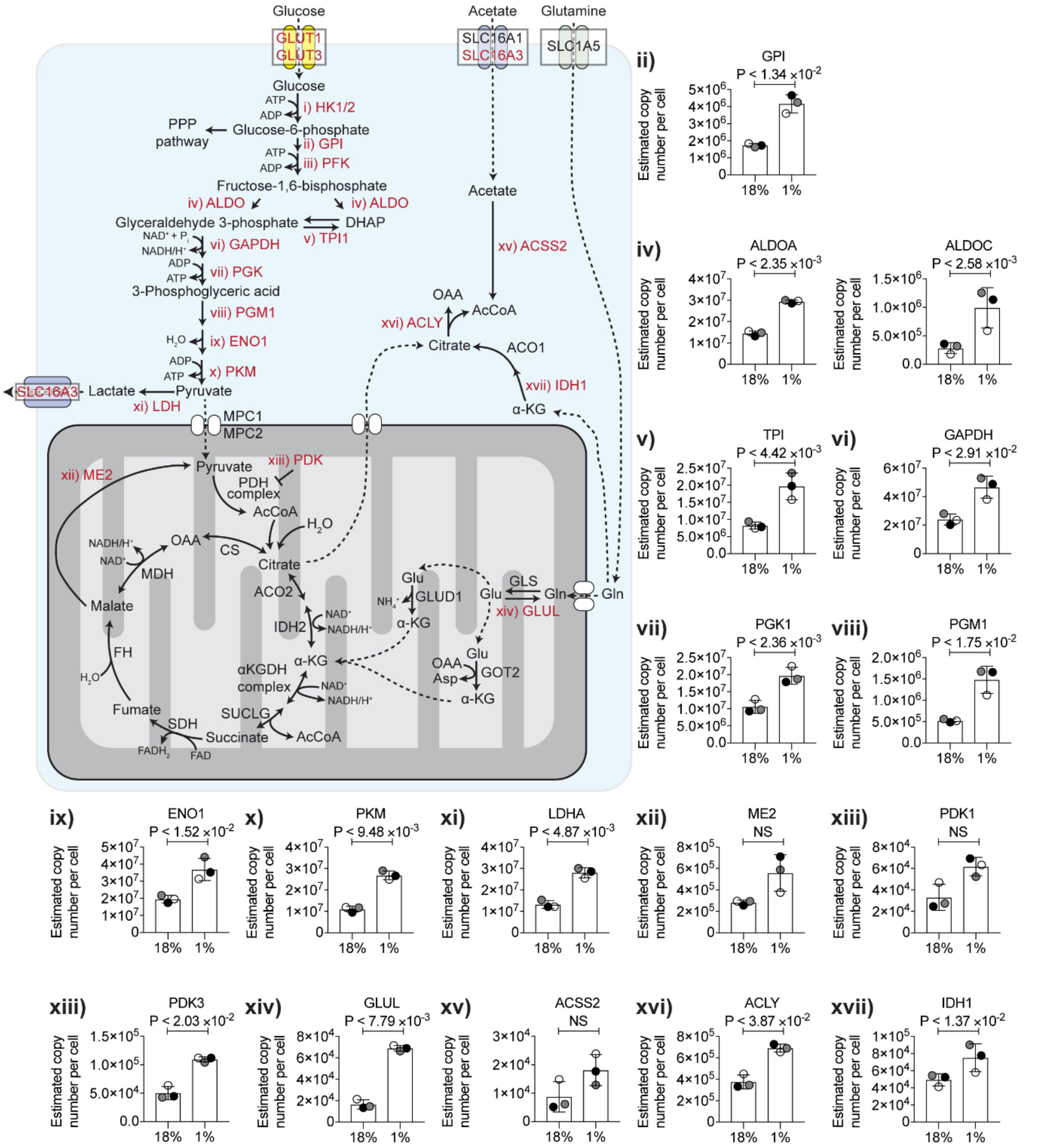

Figure S2: Impact of hypoxia on metabolic pathways linked to the TCA cycle. Schematic representation of metabolic pathways that can feed intermediates into the tricarboxylic acid (TCA) cycle. Enzymes involved in metabolic steps are indicated. Those numbered, and in red, increase in abundance with hypoxia. Copy numbers estimated from the proteomic data for these indicated proteins are shown alongside. In bar charts, data points from the three biological replicates are colour matched, the bar shows the mean and the error bars show standard deviation. P values were calculated using a two-tailed paired t-test. Non-significant changes are marked NS.

Figure S3

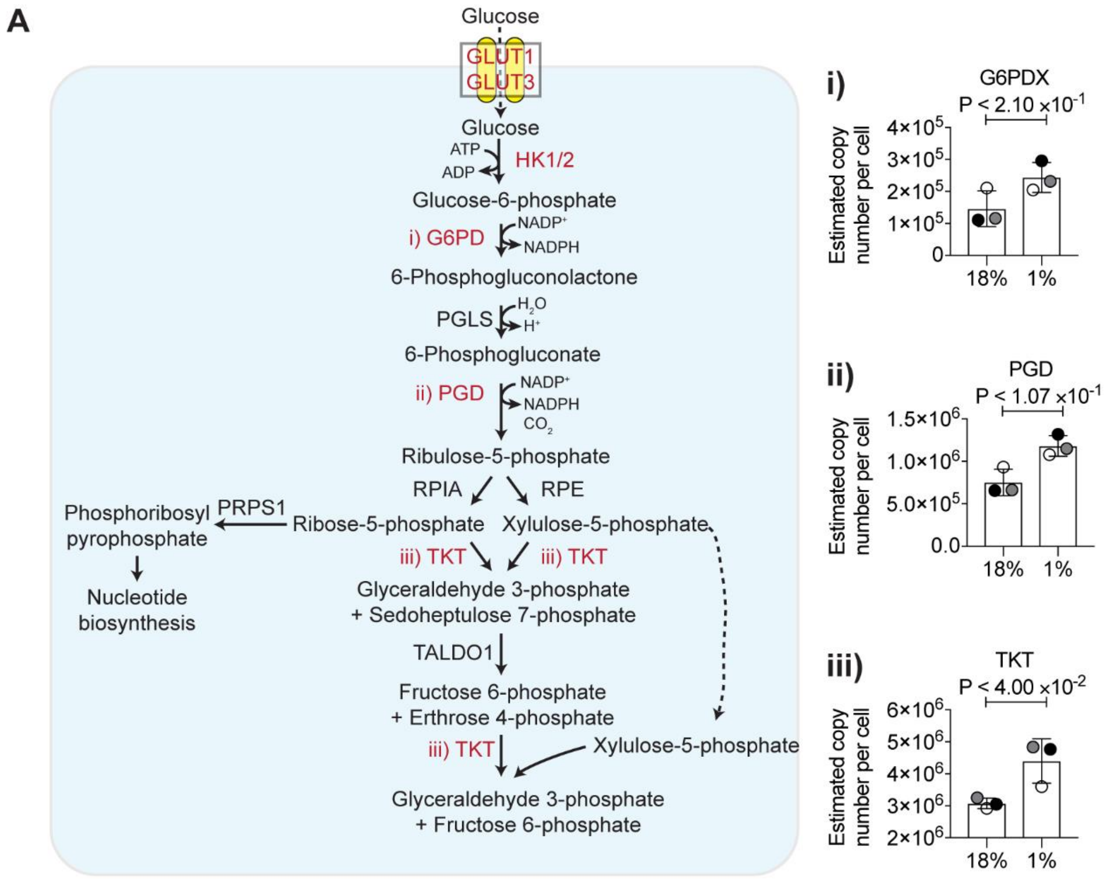

Figure S3: Impact of hypoxia on the pentose phosphate pathway

(**A**) Schematic representation of the pentose phosphate pathway (PPP) cycle. Enzymes involved in metabolic steps are indicated. Those numbered and in red, increase in abundance with hypoxia. Copy numbers estimated from the proteomic data for the indicated proteins are shown alongside. In bar charts, data points from the three biological replicates are colour matched, the bar shows the mean and the error bars show standard deviation. P values were calculated using a two-tailed paired t-test.

Figure S4

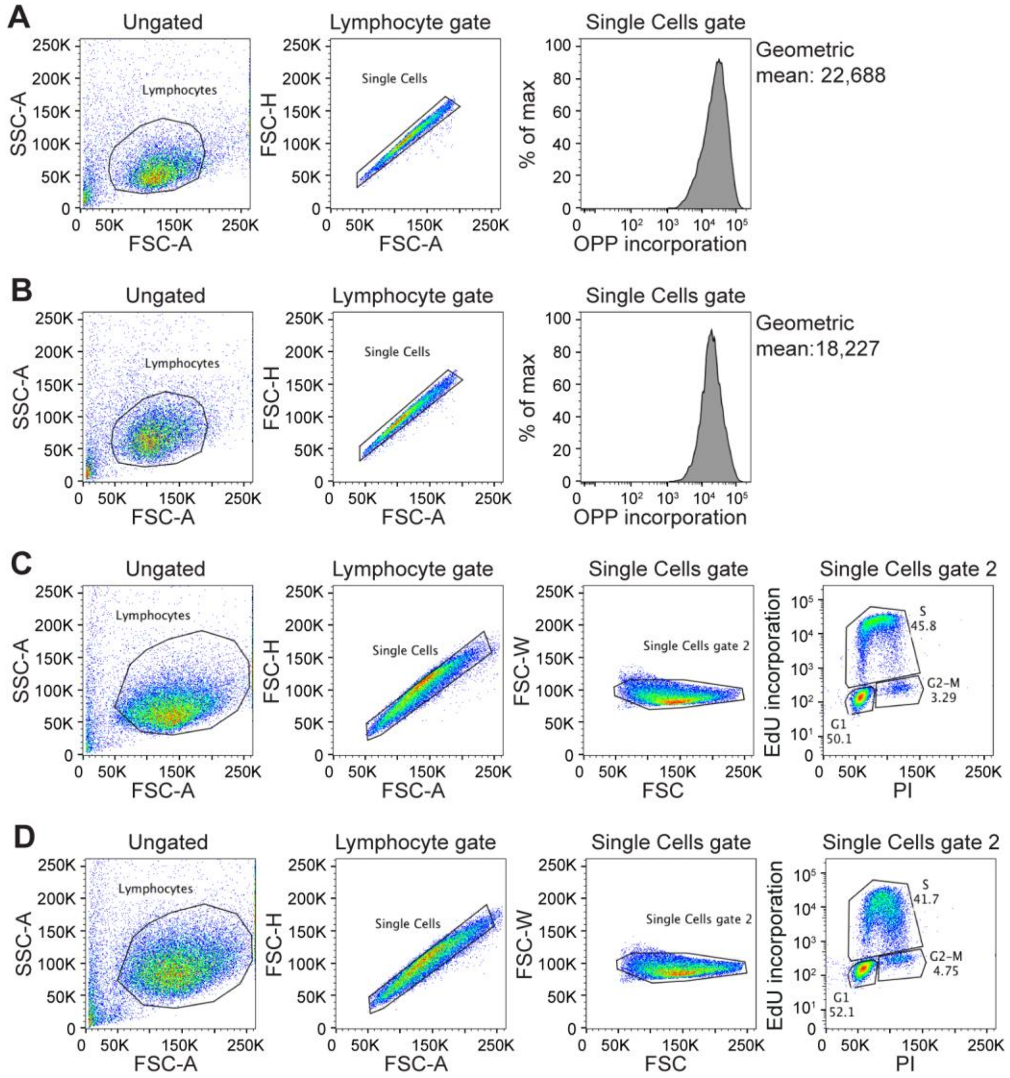

Figure S4: Gating strategies for flow cytometry assays

(**A**) and (**B**) Gating strategy for the OPP protein synthesis assay on pure cultures of CTLs in normoxia (**A**) and hypoxia (**B**) associated with Figure 3G and 3H. The geometric mean for the histograms from the Single Cells gate is shown alongside. (**C**) and (**D**) Gating strategy for the EdU DNA synthesis assay on pure cultures of CTLs in normoxia (**C**) and hypoxia (**D**) associated with Figure 4C, 4D and 4E.

Supplementary Table legends

Table S1. The proteome of normoxic and hypoxic CTL maintained in IL-2
Proteins identified in CTLs by MaxQuant analysis, excluding those identified as being known contaminants, reverse sequences and proteins only identified based on a modification site. Copy numbers per cell were calculated using the proteomic ruler plug-in in Perseus and used to calculate the ratio of protein abundance (hypoxia/normoxia). Protein ratios that increased by ≥1.5-fold are coloured yellow and those that decreased by ≥1.5-fold are highlighted in purple. The significance column shows the P values (calculated by performing a two-tailed paired Student's t-test, without further adjustment, on Log2-normalized copy numbers for each protein in normoxia and hypoxia). Perseus software was used to annotate the proteins in the dataset with GO terms and KEGG pathways. The tab "All data" shows the full data set. The "Proteins increased by hypoxia" tab shows proteins significantly increased by hypoxia (hypoxia/normoxia ratio of ≥1.5, reproducibly regulated in all replicates and with a P value of ≤0.05). The tab "Proteins decreased by hypoxia" shows proteins significantly decreased by hypoxia (hypoxia/normoxia ratio of ≤ 0.67, reproducibly regulated in all replicates and with a P value of ≤0.05). The tab "DAVID analysis" includes the list of background proteins and proteins increased in response to hypoxia used in the analysis, the settings used, and the output results. The tab "P(A)RBP hypoxic translation" shows poly(A) RNA binding proteins that have been reported to control the reprogramming of the translatome during hypoxia. For copy numbers, quantification accuracy of each protein was annotated as follows: high: ≥8 peptides detected, minimum of 75% unique peptides; medium: ≥3 peptides detected, a minimum of 50% unique peptides; low: all other peptides.

Table S2. The mass of proteins in normoxic and hypoxic CTLs
Estimated copy numbers per cell were used to calculate the mass of specific proteins, or groups of proteins, per cell, as described in "Materials and Methods". The tab "Data with mass calculations" shows the protein masses calculated for all data. The tabs "Glycolysis", "OxPhos" and "Ribosomes" show the masses of the proteins filtered using the KEGG pathway annotations "glycolysis", "oxidative phosphorylation" and ribosomes respectively. The total mass of proteins per cell, or total mass of proteins within each filtered group, was calculated, and are shown at the bottom of the protein lists.
